# US Food and Drug Administration Shifts to AI-Enhanced Regulatory Review With Elsa 4.0 and HALO

**DOI:** 10.2196/101884

**Published:** 2026-05-29

**Authors:** Tejas S Athni

**Keywords:** FDA, US Food and Drug Administration, Elsa 4.0, HALO, Harmonized AI and Lifecycle Operations for Data, generative AI, artificial intelligence, regulatory review, AI-enhanced, harmonized AI, medical devices, health policy

## Abstract

The US Food and Drug Administration recently announced the launch of Elsa 4.0 and Harmonized AI and Lifecycle Operations for Data (HALO)—an upgrade to its artificial intelligence platform. In this *News and Perspectives* article, JMIR Correspondent Tejas S Athni reports on the evolution of the Elsa model and potential benefits and challenges.

**Key Takeaways:**

- The US Food and Drug Administration’s (FDA’s) Elsa 4.0 artificial intelligence (AI) model and Harmonized AI and Lifecycle Operations for Data (HALO) platform shift AI from a peripheral tool to an embedded interface for querying, synthesizing, and acting on regulatory data across siloed systems.
- Integration with HALO may accelerate evidence synthesis and reduce review timelines, potentially enabling faster patient access to high-impact therapies and devices.
- These gains depend on robust human verification of Elsa outputs, as risks such as AI error propagation, workforce instability, and regulatory deskilling could undermine accuracy.

The US Food and Drug Administration’s (FDA’s) May 6, 2026, release of Elsa 4.0—a generative artificial intelligence (AI)–powered decision support system—marks a broader transition in how AI is deployed in federal health regulatory workflows and a shift in how FDA regulators access, synthesize, and act on data to make decisions. As an upgrade to the agency’s previous AI system, Elsa 4.0 brings new capabilities and new challenges.

## From Elsa 1.0 to 4.0

The original Elsa—Elsa 1.0—was launched in June 2025 as part of a broader modernization effort across the Department of Health and Human Services (HHS), in line with a 2025 White House directive encouraging federal agencies to adopt generative AI for efficiency. Built within a FedRAMP High–compliant Google Cloud platform environment, Elsa was designed as an internal AI assistant to aid FDA employees by reading, writing, and summarizing regulatory information. Specifically launched to support tasks such as adverse event summarization, label comparison, clinical protocol review, and identification of high-priority inspection targets, its other uses included drafting communications and generating code to build internal databases for nonclinical applications.

The goal was straightforward—accelerating evidence synthesis and cutting review times while reducing administrative burden and streamlining routine cognitive tasks. Early reactions to Elsa 1.0 suggested that it delivered operational value in streamlining routine tasks while serving as a “practical quick win” that built internal confidence in AI’s role within regulatory workflows. However, others noted that the system hallucinated, generating fabricated studies or misrepresenting research, with some FDA reviewers describing the tool as “clunky.” Subsequent versions, including Elsa 2.0 and 3.0, were rolled out over the following months with incremental upgrades in usability and scope. Elsa remained largely an adjunct tool. Staff still needed to gather and input data manually into the Elsa system. The FDA shifted the underlying model architecture from Anthropic’s Claude to Google’s Gemini.

Released in May 2026, Elsa 4.0 represents a more structural shift and a progression from task-based assistance to integrated data querying and analysis, with AI increasingly embedded into core FDA functions. It is paired with Harmonized AI and Lifecycle Operations for Data (HALO)—a platform that consolidates 40+ previously separate and siloed FDA systems into a single environment. With this integration, Elsa now sits directly atop the agency’s data; instead of assembling documents independently from multiple sources, FDA staff can query across datasets through the AI interface itself, with new functionality and improved chat flexibility. Together, this can enable FDA staff to build and execute workflows directly within the system rather than rely on individual tools. The FDA has also emphasized data security: the AI model is not trained on users’ input data or data submitted by FDA-regulated companies, addressing concerns about confidentiality and data leakage.

**Figure:**
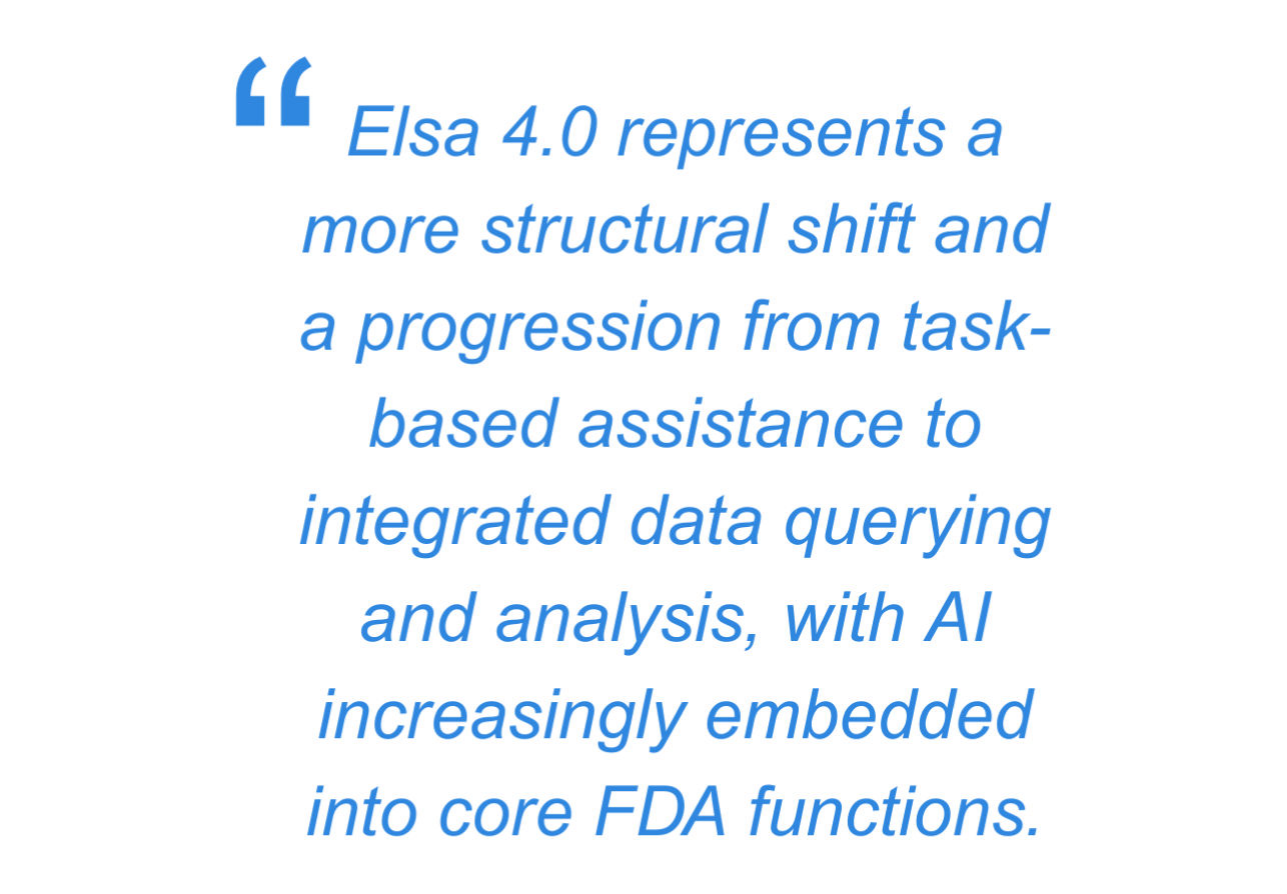


## A Subtle but Structural Transformation

Elsa 4.0 and HALO change how regulatory work unfolds at the FDA. AI is no longer a sideline assistant in the regulatory pipeline. Rather, AI can now function as a core data synthesizer that takes regulator queries to retrieve and evaluate pertinent information. Elsa 4.0’s ability to automate the generation of charts and visualizations can help reviewers more quickly interpret trends, safety signals, or comparative outcomes. Similarly, optical character recognition capabilities allow legacy files to become fully searchable, reducing the risk of relevant information remaining buried in nonindexed formats. Voice-to-text dictation can enable regulatory reviewers to capture notes and observations in real time without interrupting their analytic workflow. Elsa 4.0 repositions AI in the regulatory pipeline from a modular tool to an increasingly central, overlying interface. Just as importantly, this means that human reviewers must be in the loop to verify and contextualize results, even if the first pass is made by generative AI.

For the public and patients, the effects of the Elsa 4.0 and HALO shift are unlikely to be immediately visible. Regulatory decisions will still follow established standards, and drug and device approvals will not simply be dictated by an automated AI decision output. However, the impact will likely emerge through improvements in how efficiently regulatory work can be performed and reduced frictions. For instance, within the FDA Breakthrough Devices Program, a reviewer evaluating a novel implantable surgical device through the premarket approval pathway may need to synthesize evidence across prior internal submissions, 510(k) clearances for related or predicate devices, pivotal clinical evidence generated under investigational device exemption studies, and postmarket safety signals in the Manufacturer and User Facility Device Experience (MAUDE) adverse event reporting database. Traditionally, this requires navigating multiple internal data systems to retrieve documents. With HALO integration, the reviewer can query across these datasets simultaneously, potentially reducing the timeline of task-specific work. This could shorten the overall time to regulatory decisions, allowing high-impact devices to reach patients sooner. In principle, even modest reductions in review timelines may translate into earlier access to novel therapies without compromising the underlying rigor of evaluation.

## Risks and Early Lessons

The upgrade is, however, not without potential risks. With a unified HALO data platform, errors in retrieval or synthesis are no longer confined to a single query; they can propagate across the review pipeline, much like the swarm-like amplification of errors seen in AI-to-AI interactions. These potential errors can require additional time to verify and correct the information, which poses a risk to the efficiency gains promised by AI and could lengthen—not shorten—review timelines. This tension is compounded by the risk of sudden workforce reductions. The HHS terminated approximately 3500 FDA employees in April 2025 alone, before beginning partial rehiring in May 2025. These abrupt cuts create a standing risk that human verification capacity may suddenly be diminished, leaving Elsa 4.0 deployed into an agency that may not consistently be able to rigorously validate its AI outputs.

Regulatory deskilling is also a concern, as repeated reliance on AI for tasks such as synthesis and analysis may reduce human reviewers’ direct engagement with primary evidence. Gradually, this can lead to the erosion of core evaluative skills, the weakening of domain judgment, and reduced ability to independently flag errors. The United States also lacks a unified regulatory structure for AI use in settings like health care and medical devices, which places the FDA in the position of both adopting AI internally and defining its best practices in real time. These challenges suggest that successful AI integration in the FDA will depend not only on technical capability but, as I’ve previously written, also on leadership structures and an agency-wide organizational culture that can sustain reviewer expertise and human-in-the-loop governance.

